# Insect responses to host plant provision beyond natural boundaries: latitudinal and altitudinal variation in a Chinese fig wasp community

**DOI:** 10.1002/ece3.1622

**Published:** 2015-08-13

**Authors:** Rong Wang, Stephen G Compton, Rupert J Quinnell, Yan-Qiong Peng, Louise Barwell, Yan Chen

**Affiliations:** 1 Ecological Security and Protection Key Laboratory of Sichuan Province, Mianyang Normal UniversitySichuan, 621000, China; 2 School of Ecological and Environmental Sciences, Tiantong National Station of Forest Ecosystem, East China Normal UniversityDongchuan Road 500, Shanghai, 200241, China; 3 School of Biology, University of LeedsLeeds, LS2 9JT, UK; 4 Department of Zoology & Entomology, Rhodes UniversityGrahamstown, 6140, South Africa; 5 Key Laboratory of Tropical Forest Ecology, Xishuangbanna Tropical Botanical Garden, Chinese Academy of SciencesKunming, 666303, China

**Keywords:** Agaonidae, climate tolerance, *Ficus microcarpa*, gall, latitudinal gradient, parasitoid, trophic level.

## Abstract

Many plants are grown outside their natural ranges. Plantings adjacent to native ranges provide an opportunity to monitor community assembly among associated insects and their parasitoids in novel environments, to determine whether gradients in species richness emerge and to examine their consequences for host plant reproductive success. We recorded the fig wasps (Chalcidoidea) associated with a single plant resource (ovules of *Ficus microcarpa*) along a 1200 km transect in southwest China that extended for 1000 km beyond the tree's natural northern range margin. The fig wasps included the tree's agaonid pollinator and other species that feed on the ovules or are their parasitoids. Phytophagous fig wasps (12 species) were more numerous than parasitoids (nine species). The proportion of figs occupied by fig wasps declined with increasing latitude, as did the proportion of utilized ovules in occupied figs. Species richness, diversity, and abundance of fig wasps also significantly changed along both latitudinal and altitudinal gradients. Parasitoids declined more steeply with latitude than phytophages. Seed production declined beyond the natural northern range margin, and at high elevation, because pollinator fig wasps became rare or absent. This suggests that pollinator climatic tolerances helped limit the tree's natural distribution, although competition with another species may have excluded pollinators at the highest altitude site. Isolation by distance may prevent colonization of northern sites by some fig wasps and act in combination with direct and host-mediated climatic effects to generate gradients in community composition, with parasitoids inherently more sensitive because of declines in the abundance of potential hosts.

## Introduction

The spatial distributions of species reflect the net effects of numerous historical, geographic, biotic, and abiotic elements including speciation, migration, species interaction, resource availability, and climatic tolerances (Gaston 2000; He et al. 2005; Chen and He 2009). Phytophagous insects are the most species-rich components of terrestrial communities. The distributions of specialist phytophages are necessarily limited to within the ranges of their host plants, but they often only occupy a proportion of their host's range, suggesting that additional physical and biological variables such as climate and natural enemies also routinely play a part in determining range boundaries (Strong et al. 1984). Temperatures, precipitation, and the extent of seasonal fluctuations in climate all change with latitude and elevation (Hodkinson 2005; Deutsch et al. 2008; Benton 2009; Feeley et al. 2012; Yasuhara et al. 2012), and host plants can often tolerate a wider range of temperatures than their associated insects. The insect community associated with bracken provides an example, with some bracken-feeding species restricted to frost-free areas in Africa (Compton et al. 1989). This results in fewer insect associates at higher latitudes and altitudes and contributes to the all-pervading latitudinal gradient in species diversity (Gaston 2000; Willig et al. 2003; Witman et al. 2004; Buckley et al. 2010). The distributions of insects may nonetheless respond differently and more rapidly to changes in climate than their hosts (Schönrogge et al. 2012; Nooten et al. 2014), resulting in the proportion of a host plant's range that is utilized changing over time (Hodkinson and Bird 1998; Schweiger et al. 2008).

Gradients in altitude display some environmental changes that are similar to latitudinal gradients, and can generate similar selection pressures on phytophagous insects (Pellissier et al. 2012), but there are also major differences that can influence how insects respond (Hodkinson 2005). Altitudinal effects are typically displayed over much shorter distances, making dispersal between sites with contrasting temperature regimes easier than between equivalent climatic changes generated by latitude. Seasonal variation in temperatures and day length are also lower at high elevations at lower latitudes, compared with lower elevation, higher latitude sites. Challenges posed to phytophagous insects and their host plants at higher latitudes are therefore similar, but not the same as those generated at higher altitudes (Rasmann et al. 2014).

Phytophagous insects often support diverse communities of parasitoids, many of which also have restricted host ranges. The distributions of carnivores and others that feed at higher trophic levels often decline rapidly with latitude (Hillebrand 2004; Freestone et al. 2011; Santos and Quicke 2011) and suitable hosts may be less abundant or entirely absent at higher latitudes for parasitoids with highly specific host requirements (Condamine et al. 2012; Cruaud et al. 2012). Similarly, individuals of a phytophagous species that develop at higher altitudes can be subject to attack by fewer species of parasitoids, and suffer lower mortality rates (Randall 1982).

Phylogeographic history and interactions within complex large-scale communities often make the drivers of insect distributions and responses to environmental gradients difficult to distinguish (Buckley et al. 2010; Romdal et al. 2013). Relatively simple spatially defined insect communities associated with a single plant resource provide good systems to try to tease apart drivers of species distribution (Hawkins and Compton 1992; Bannerman et al. 2012). Large-scale manipulations of these communities result when the distributions of their host plants are extended beyond their natural range margins, especially where the expanded range extends into areas subject to more extreme environmental conditions. Here, we focus on a community of plant-feeding and parasitoid fig wasps (Hymenoptera, Chalcidoidea) associated with a single plant resource – the figs produced by a species of fig tree that is widely planted within and beyond its natural distribution in China.

The obligate mutualism between fig trees (*Ficus* species, Moraceae) and their pollinating fig wasps (Chalcidoidea, Agaonidae) is highly taxon specific, with most of the more than 800 species of fig trees pollinated by one or a small number of fig wasps (Wiebes 1979; Herre et al. 2008; Chen et al. 2012). Related fig trees tend to be pollinated by related fig wasps, suggesting a long history of co-evolution, although host switching between lineages has also taken place (Cook and Segar 2010; Cruaud et al. 2012). The mutualism is also of broader ecological significance, because so many vertebrates eat ripe figs, resulting in fig trees and fig wasps often being keystone species (Shanahan et al. 2001; Herre et al. 2008). Most fig trees have tropical or subtropical distributions, and few species are exclusively temperate. Factors influencing their range margins may be linked to the trees themselves or reflect limitations imposed by the environmental tolerances of their pollinators (Liu et al. 2014; Zhang et al. 2014).

Figs are also utilized by other groups of fig wasps belonging to families of Chalcidoidea other than Agaonidae. More than 30 nonpollinating fig wasps (NPFW) species have been recorded from a single *Ficus* species, although most support less than half this number (Compton and Hawkins 1992; Cook and Rasplus 2003; Wang et al. in press). Like the pollinators, NPFW generally develop in galled ovules. Figs lacking pollinators usually abort, but some NPFW are capable of developing in unpollinated figs, which allows them to be independent of the pollinators. The host ranges of most NPFW are poorly known. Some lineages appear highly host plant specific, but others contain species that utilize more than one host plant (Cook and Segar 2010). Detailed knowledge of the larval feeding behavior of NPFW is only available for a small number of species, but it is becoming increasingly apparent that NPFW display a diverse range of feeding behaviors which includes seed predators, ovule and fig wall primary gallers, secondary gallers that enlarge the galls of primary gallers, primary parasitoids (most of which also feed on some plant tissue), and specialist hyperparasitoids (Pereira et al. 2007; Compton et al. 2009; Segar and Cook 2012; Chen et al. 2013). The specific insect hosts attacked within figs by parasitoid NPFW are rarely documented (Cook and Segar 2010; Segar et al. 2013), but niche conservatism induced by morphological characters such as ovipositor length and body size generates some specific matching of parasitoids and gallers, and there is also evidence for strict sense co-evolution between some gall-formers and their specific parasitoids (Compton 1993; West et al. 1996; Dunn et al. 2008). Because of this limited knowledge, it is usually only possible to characterize the species within a particular fig wasp community as being either exclusively phytophages (most or all of which are ovule gall-formers) and parasitoids in a broad sense, which kill larvae of other fig wasps and develop in galls that other species had initiated.

Fig wasp communities display convergence and relatively homogeneous structures across continents (Segar et al. 2013), but also display between-site variation in species richness. Latitudinal gradients in the species richness and composition of fig wasp faunas in southern Africa have been investigated along a gradient extending from six degrees north to 34 degrees south (Compton and Hawkins 1992; Hawkins and Compton 1992). These studies failed to detect significant latitudinal trends in the species richness of galler NPFW, whereas species richness among parasitoid fig wasps was generally slightly lower at lower latitudes, but for most species, only a small number of crops were available for analysis. Here we focus on geographic variation in the community of fig wasps associated with the figs of a single species of Asian fig tree *Ficus microcarpa*. We recorded the fig wasp communities associated with *F. microcarpa* along a 1200 km roughly north–south transect that extended northwards from within the plant's native range. *Ficus microcarpa* is one of the most widely planted street trees in southern China, both within and beyond its natural range. We recorded the composition of its fig wasp fauna and focused on the following questions: (1) How does the fig wasp community vary at different altitudes, latitudes, and distances from the natural range? (2) Are climatic factors correlated with community composition? (3) Do species feeding at different trophic levels respond differentially to latitude and its associated climate factors? and (4) Do changes in fig wasp community composition contribute to range boundary determination in this fig tree?

## Materials and Methods

### *Ficus microcarpa* and its fig wasps

**Figure 1:**
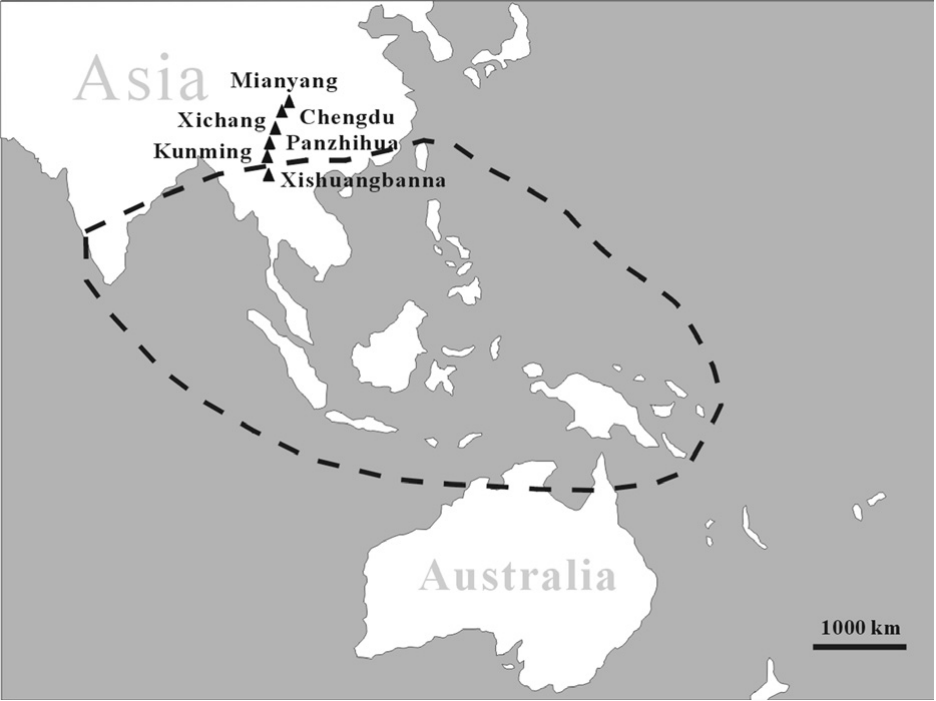
The natural distribution range of *Ficus microcarpa* and the sample sites in the present study.

*Ficus microcarpa* L. is a monoecious fig tree with a natural distribution that extends from India and southern China to northern Australia (Berg and Corner 2005; Fig.1). As a result of its popularity as a street and ornamental tree, and the widespread introduction of its pollinators, *F. microcarpa* populations have become established in many tropical and subtropical areas, including the Mediterranean and Caribbean, mainland USA and Hawaii and Brazil (Nadel et al. 1992; de Figueiredo et al. 1995; Kobbi et al. 1996; Beardsley 1998; Burrows and Burrows 2003; Starr et al. 2003; van Noort et al. 2013; Wang et al. 2015). In urban environments, small plants can cause damage to buildings, but the plant can also become a serious invader of natural habitats (Mckey 1989; Beardsley 1998; Starr et al. 2003; Corlett 2006; Caughlin et al. 2012). The plant's success in seasonal climates may be related to the ability of its pollinators’ populations to rapidly recover from winter shortages of figs (Yang et al. 2013). *F. microcarpa* has small seeds that are mainly dispersed by frugivorous birds, and ants also serve as secondary dispersal agents (Kaufmann et al. 1991; Shanahan et al. 2001; Caughlin et al. 2012).

In China, *F. microcarpa* is indigenous to south Fujian, Guangdong, Guangxi, Hainan, the south of Yunnan Province and Taiwan. It is also one of the most widely planted ornamental and street trees in southern China, both within its natural range and extending to around 1000 km further north. Within its natural range, *F. microcarpa* is generally an uncommon component in natural forests. It is present at much higher densities in urban or suburban areas and we mainly focused on the fig wasp communities found on planted trees. *F. microcarpa* produces crops of up to several thousand figs from among the axils of leaves. Fruiting is asynchronous at the population level, with figs present on the trees throughout the year. On individual trees, the crops may develop synchronously, but when few fig wasps are present, the synchrony tends to break down. Low winter temperatures in the introduced range of the plant restricts the emergence of adult fig wasps from their natal figs to the warmer months of the year, whereas emergence is all year at our southern-most sites, with a peak during the cool dry months of late winter (Y.-Q. Peng, pers. comm.). Numerous varieties and forms of *F. microcarpa* have been described within its extensive range (Berg and Corner 2005). The trees in southwest China are uniform in appearance, but could not be assigned to a particular variety.

At least 30 species of fig wasps have larvae that develop inside the figs of *F. microcarpa* (Bouček 1988; Chen et al. 1999; Feng and Huang 2010; Wang et al. 2015). Most of these fig wasps have *F. microcarpa* as their only host plant, but some are also associated with other related fig trees (Zhou et al. 2012). *F. microcarpa* is pollinated by *Eupristina verticillata* Waterston (Agaonidae), a taxon that molecular data suggest may consist of a complex of morphologically similar species (Sun et al. 2011), although in southwest China, only one species has been recorded (J.-Y. Rasplus & A. Cruaud, Pers. Comm.). In addition, *F. microcarpa* is one of the very few species of fig trees that supports an agaonid “cheater” (*Eupristina sp*. *indesc.)* that fails to pollinate its host figs (J.-Y. Rasplus, Pers. Comm.). Adult females of the agaonids enter *F. microcarpa* figs in order to lay their eggs into the ovules via the styles of the flowers. Both agaonids have larvae that develop inside galled ovules and feed exclusively on plant tissue.

All the known fig wasps associated with *F. microcarpa* figs utilize its ovules for larval development. Unlike agaonids, the NPFW lay their eggs into these ovules via the outer wall of the fig, utilizing their very long ovipositors (Galil and Copland 1981). As with the agaonids, a single larva develops inside each ovule. Parasitoid NPFW may or may not consume some plant tissue, but they always destroy the gall causers. Some gallers of *F. microcarpa* ovules can develop in figs that are not entered by pollinators. They have associated parasitoids that do not attack pollinator larvae (S.G. Compton & R. Wang, unpubl.). Generally, species from subfamily Epichrysomallinae (family Pteromalidae) are the hosts of species from family Eurytomidae, and species from subfamily Sycoryctinae (family Pteromalidae) are parasitoids of both agaonids and species from subfamily Otitesellinae (family Pteromalidae). *Philotrypesis taiwanensis* Chen (Sycoryctinae) is the only obligate seed predator (Wang et al. 2014). In our analyses, we grouped the species associated with *F. microcarpa* into two trophic levels based on their feeding behavior: “phytophages” with larvae that feed exclusively on plant ovules or seeds and “parasitoids” with larvae that kill other fig wasp species.

### Study sites

*Ficus microcarpa* fig crops were sampled in Mianyang, Chengdu, Xichang, and Panzhihua (Sichuan Province), and Kunming and Xishuangbanna (Yunnan Province) (Fig.1). They formed a north–south transect across southwestern China, covering about 1200 km and 9.5 degrees of latitude. Xishuangbanna is located on the border between subtropical and tropical China, with hot and humid summers and mild, dry winters. It is the only study site believed to be within the native range of *F. microcarpa* (Table1). At the other sites, *F. microcarpa* is not present in local natural forests, but has been widely planted in urban areas. Winter and summer temperatures at the sites generally decline with increasing latitude, but Kunming has a cooler climate than the other sites, because of its higher elevation (Table1). Variation in annual precipitation among the study sites is slight, ranging from 850 to 1100 mm. The trees in Xishuangbanna were growing in a botanic garden. Elsewhere, they were planted along roadsides and in public amenity areas.

**Table 1:** Locations and collection dates of *Ficus microcarpa* fig samples and the proportions of the figs that had been colonized by fig wasps. Study sites are ordered from north to south. Meteorological data for the period 2004–2013 were obtained from the website of Weather Underground (http://www.wunderground.com)

Study site	Location	Altitude (m)	Annual precipitation (mm)	Mean monthly minimum (^o^C)	Mean monthly maximum (^o^C)	Sampling dates	*N* crops	Crops with fig wasps	*N* figs	Occupied figs (%) (mean ± SE)
Mianyang	N 31° 28′, E 104° 41′	460	927.5	12.9	20.8	July 2012	15	5	491	9.10 ± 4.77
Chengdu	N 30° 40′,E 104° 06′	500	865.5	13.1	20.3	July 2012	6	3	77	24.37 ± 11.03
Xichang	N 27° 53′,E 102° 17′	1533	1013.6	12.3	23.1	July 2012	14	12	277	60.62 ± 8.21
Panzhihua	N 26° 35′,E 101° 43′	1150	NA	NA	NA	July 2012	10	9	155	74.99 ± 9.81
Kunming	N 24° 53′,E 102° 50′	1891	1011.2	10.3	20.8	July 2011	6	5	42	79.63 ± 16.33
Xishuangbanna	N 22° 00′,E 100° 48′	553	1113.6	18.0	29.6	Dec. 2010- Jan. 2012	12	12	164	98.85 ± 0.82
Overall	–	–	–	–	–	–	63	46	1206	56.27 ± 5.20

NA, not available.

### Collecting methods

We haphazardly collected mature figs without fig wasp exit holes (late C/early D) phase *sensu* Galil and Eisikowitch (1968) from at least six *F. microcarpa* trees at each study site (Table1) and stored them in 70% ethanol. Sampling was concentrated in the periods when most trees locally had mature figs. Figs that are not colonized by fig wasps are retained on the trees for long periods before they abort. They continue to grow and could only be reliably distinguished from figs entered by fig wasps after dissection. To record the contents of the figs, they were cut into quarters and soaked in water for at least 10 min to soften the galls before the figs were examined under a binocular dissecting microscope. Each flower was checked and recorded in one of the following categories: male flowers, seeds, unfertilized and ungalled female flowers, galls containing wasps, and failed galls. Failed galls (“bladders”) were hollow or contained the remains of dead fig wasp larvae. The fig wasps were identified as morpho-species mainly based on Chen et al. (1999) and Feng and Huang (2010) using the classification of Rasplus et al. (1998), Campbell et al. (2000), Cruaud et al. (2010), and Heraty et al. (2013) as shown in figweb (http://www.figweb.org).

### Statistical analyses

All statistical analyses except species accumulation and estimated species richness curves were carried out using R version 2.14.2 (R Development Core Team 2012). Likelihood ratio tests were carried out to assess the significance of fixed effects. The effect of latitude on the presence or absence of any fig wasps in the figs was analyzed using a generalized linear model (GLM) assuming a quasibinomial distribution of residuals. Only figs that contained fig wasps were used in further analyses.

We tested whether we had detected most or all of fig wasp species in their regional species pools by delineating curves of accumulated species richness with increasing sample size using a first-order jackknife algorithm (Burnham and Overton 1978; Heltshe and Forrester 1983), in SDR version 4.1.2 (Seaby and Henderson 2006).

The impacts of latitude on female flower number per fig, the numbers of total and female pollinator adult offspring (the plant's male reproductive function), and the numbers of seeds (the plant's female reproductive function) per fig were assessed using GLMs with quasi-Poisson distribution of residuals.

Meteorological data for the period 2004–2013 were obtained from the website of Weather Underground (http://www.wunderground.com). For each study site, except Panzhihua, we collated eight climate factors: annual average temperature and precipitation, summer (3 months from June to August) average and average high temperatures, summer average rainfall, winter (3 months from December to February) average and average low temperatures, and winter average rainfall. Panzhihua was excluded from the climate analyses due to a lack of local meteorological data. We then ran a principal component analysis (PCA) and selected the first principal component (PC1, which explained 61.1% of the total variance) as the factor representing “local climate” (Table S1 in supplementary materials).

Because of the strong correlation between the altitude and local climate (PC1) at our study sites (Pearson's product–moment correlation: altitude vs. local climate: *r* = 0.880, *t* = 3.214, df = 3, *P* = 0.046; altitude vs. latitude: *r* = −0.131, *t* = 0.265, df = 4, *P* = 0.804; local climate vs. latitude: *r* = 0.322, *t* = 0.590, df = 3, *P* = 0.597), we examined the influence of latitude and altitude on fig wasp community composition. The effects of those two factors (including the interaction between them) on fig wasp ovule occupancy rates within figs (the proportion of ovules that supported fig wasp adult offspring), fig wasp abundance (total number of fig wasps per fig), and species richness (total species, phytophages, and parasitoid species richness) were evaluated by GLMs assuming either quasibinomial or quasi-Poisson distribution of residuals. The trends in species richness at the two different trophic levels (for interactions between trophic level with latitude and altitude) were then compared using GLMs assuming quasi-Poisson distribution of residuals. The impacts of latitude and altitude on Shannon–Wiener index values per fig were analyzed with linear models (LMs).

## Results

### Fig utilization by fig wasps

**Figure 2:**
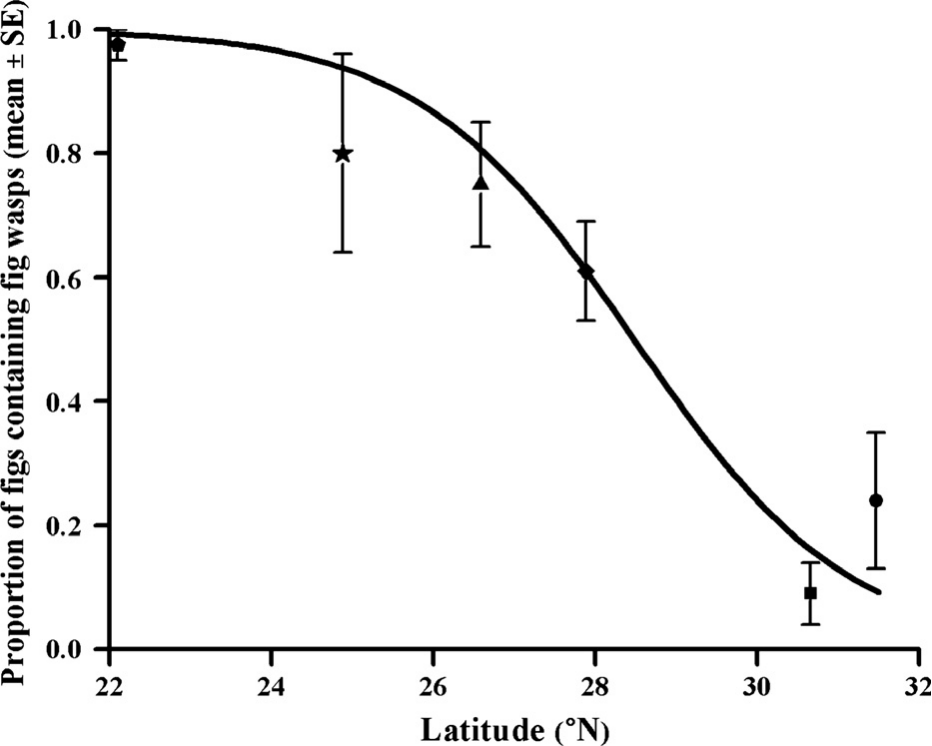
Generalized linear relationship between the proportion of figs containing fig wasps in each crop and the latitudes of study sites. A quasibinomial distribution of residuals was applied. Pentagons, stars, triangles, diamonds, squares, and circles indicate Xishuangbanna, Kunming, Panzhihua, Xichang, Chengdu, and Mianyang, respectively, ordered from south to north.

A total of 1206 mature or aborting figs were dissected from 63 crops, of which only 555 figs from 46 crops contained fig wasps (Table1). More than 60% of the figs from the more southerly areas contained fig wasps, compared with <25% of the figs in the northerly Chengdu and Mianyang collections, where the crops with no occupied figs were mainly located (Table1). The proportion of figs lacking fig wasps increased significantly at higher latitudes (GLM: â = −0.755 ± 0.103, df = 1, LR = 732.18, *P *<* *0.001; Fig.2).

### The contents of figs

At most sites, the figs contained around 160–210 female flowers (the potential oviposition sites for fig wasps). The figs from Mianyang, the most northerly site, were an exception and contained far fewer flowers of both sexes than figs elsewhere, because a large proportion of these figs were colonized by *Meselatus bicolor*, a species that galls the figs early in their development and inhibits normal floral development. However, when figs occupied by *M. bicolor* were excluded, female flower numbers per fig still declined slightly with increasing latitude (GLM: excluding figs occupied by *M. bicolor*: â = −0.029 ± 0.004, df = 1, LR = 629.26, *P* < 0.001).

**Figure 3:**
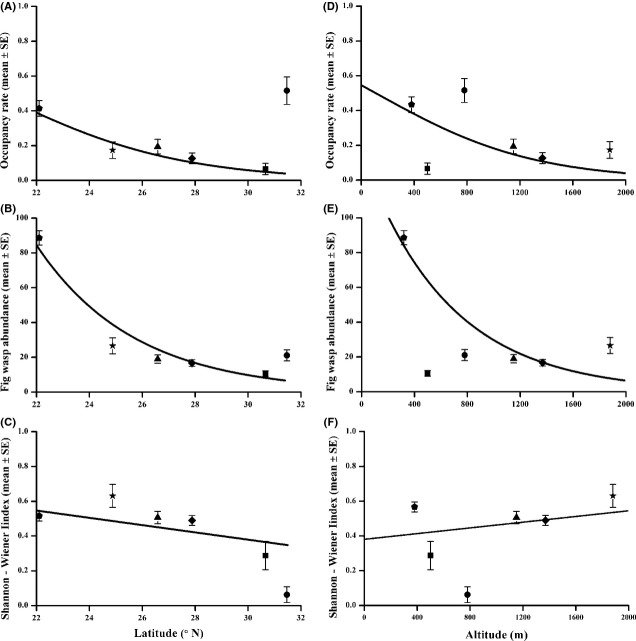
The relationships of *F. microcarpa* fig wasp community characteristics with latitude (A–C) and altitude (D–F). Only figs where fig wasps were present are considered. Ovule occupancy rates are the proportion of female flowers that contained fig wasp adult offspring. Occupancy rates and fig wasp abundance (per fig) were analyzed using GLMs assuming either quasibinomial or quasi-Poisson distribution of residuals. Shannon–Wiener index was analyzed using LMs. Pentagons, stars, triangles, diamonds, squares, and circles indicate Xishuangbanna, Kunming, Panzhihua, Xichang, Chengdu, and Mianyang, respectively (ordered from south to north).

Within the figs that had been colonized by fig wasps, ovule occupancy by fig wasp adult offspring was unusually high in Mianyang, because a majority of the figs containing fig wasps had been colonized by *M. bicolor* and this species inhibits the development of unoccupied flowers (Table2). Elsewhere, ovule occupancy was <25%, with the exception of Xishuangbanna, the most southerly site, where the figs contained far more fig wasps in total than elsewhere (Table2). Ovule occupancy and fig wasp abundance per fig declined significantly with increasing latitude (Tables2 and 3; Fig.3A,B).

**Table 2:** The contents of *Ficus microcarpa* figs (Means ± SE per fig). Only figs containing fig wasps are included. Occupancy rates are the percentage of female flowers that were occupied by fig wasps. S (obs) = total number of species recorded at the site, S (est) is the estimated total number of species present at the site, based on a first-order jackknife algorithm. H is the Shannon–Wiener diversity index

Study site	*N* crops	*N* figs	Male flowers	Female flowers	Occupancy rates (%)	Fig wasp numbers	Species richness per fig	S (obs)	S (est)	H
Mianyang	5	33	2.6 ± 1.0	43.0 ± 6.6	61.6 ± 4.9	21.1 ± 2.2	1.09 ± 0.05	2	2	0.04 ± 0.03
Chengdu	3	20	15.6 ± 1.0	166.5 ± 4.4	6.6 ± 1.2	10.6 ± 1.6	1.55 ± 0.17	5	7	0.24 ± 0.08
Xichang	12	181	16.7 ± 0.4	173.1 ± 3.5	12.7 ± 1.1	16.7 ± 0.9	2.02 ± 0.06	9	9	0.49 ± 0.03
Panzhihua	9	127	12.4 ± 0.6	167.9 ± 6.8	19.4 ± 2.2	19.0 ± 1.4	2.13 ± 0.09	12	13	0.51 ± 0.04
Kunming	5	33	17.0 ± 2.0	181.9 ± 11.5	17.4 ± 2.8	26.6 ± 3.6	2.55 ± 0.16	10	12	0.63 ± 0.07
Xishuangbanna	12	161	16.3 ± 0.6	211.1 ± 3.6	41.4 ± 1.1	88.6 ± 3.1	2.75 ± 0.11	13	14	0.48 ± 0.03
Overall	46	555	14.8 ± 0.3	175.0 ± 2.8	25.5 ± 1.0	38.7 ± 1.7	2.22 ± 0.05	21	22	0.46 ± 0.02

**Table 3:** Linear and generalized linear models examining aspects of fig wasp community composition in relation to latitude and altitude (only figs that contained fig wasps are included). Note that we did not find any significant effect of the interaction between latitude and altitude (Table S3), and therefore, it was deleted from all statistical models and the two factors were analyzed separately. LR = likelihood ratio. Response variables are as follows: (1) occupancy rates, calculated as the proportion of female flowers that supported fig wasp adult offspring, with or without inclusion of figs that contained *Meselatus bicolor* (MB), (2) fig wasp abundance (the numbers of wasps present in figs occupied by fig wasps), (3) Shannon–Wiener diversity index (S-W index), (4) fig wasp species richness (SR), (5) phytophagous fig wasp species richness (GSR), (6) parasitoid fig wasp species richness (PSR), (7) the interaction between species richness per fig and trophic level (phytophagous vs. parasitoid species) (SR × Trophic level)

Fixed effect	Response variables	Model	Residuals distribution	Slope/â (mean ± SE)	df	LR
Latitude	Occupancy rate	GLM	Quasibinomial	−0.2896 ± 0.0198	1	9892.50***>
	Occupancy rate (no MB)	GLM	Quasibinomial	−0.3512 ± 0.0146	1	12853.00***
	Fig wasp abundance	GLM	Quasibinomial	−0.2696 ± 0.0103	1	12020.00 ***
	SR	GLM	Quasibinomial	−0.0659 ± 0.0067	1	45.87***
	S-W index	LM	Normal	−0.0215 ± 0.0056	1	14.77***
	GSR	GLM	Quasi-Poisson	−0.0507 ± 0.0063	1	21.05***
	PSR	GLM	Quasi-Poisson	−0.1240 ± 0.0201	1	35.02***
	SR × Trophic level	GLM	Quasi-Poisson	–	1	9.52***
Altitude	Occupancy rate	GLM	Quasibinomial	−0.0016 ± 0.0001	1	8932.70***
	Occupancy rate (no MB)	GLM	Quasibinomial	−0.0018 ± 0.0001	1	10436.00***
	Fig wasp abundance	GLM	Quasibinomial	−0.0015 ± 0.0001	1	9943.70***
	SR	GLM	Quasibinomial	−0.0002 ± 0.0001	1	7.08***
	S-W index	LM	Normal	0.0001 ± 0.0001	1	5.44*
	GSR	GLM	Quasi-Poisson	−0.0002 ± 0.0001	1	8.49***
	PSR	GLM	Quasi-Poisson	−0.0001 ± 0.0001	1	1.25^NS^
	SR × Trophic level	GLM	Quasi-Poisson	–	1	0.14^NS^

NS, not significant, *P < 0.05; ***P < 0.001.

Only 24.5% of the 555 figs with fig wasps contained any adult offspring of pollinating agaonids, with pollinator prevalence ranging from zero in Mianyang and Kunming to almost 70% in Xishuangbanna. The mean abundance of pollinator adult offspring in the figs where they were present also varied greatly, from about six at Chengdu to over 32 per fig at Xishuangbanna (Table4). Pollinator sex ratios were female biased, with about four female pollinator offspring per fig in Chengdu and 29 in Xishuangbanna (Table4). The proportion of figs that contained seeds followed the same patterns as with pollinator offspring, with 30% or less of the figs with fig wasps containing any seeds, except at Xishuangbanna (Table4). Seed numbers in figs that contained seeds was also highly variable between sites, varying from only about two seeds per fig in Kunming, to around 29 seeds per fig at Xishuangbanna. Total and female *E. verticillata* adult offspring and seed numbers declined with increasing latitude (GLMs: pollinator: â = −0.527 ± 0.058, df = 1, LR = 6093.30, *P* < 0.001; female pollinator: â = −0.563 ± 0.061, df = 1, LR = 5759.10, *P* < 0.001; seeds: â = −0.539 ± 0.060, df = 1, LR = 5409.00, *P* < 0.001).

**Table 4:** Prevalence and abundance of two agaonid species and seeds (only figs that contained fig wasps are considered). Prevalence is the percentage of figs containing particular species or seeds. Males of pollinators could not be distinguished from those of a nonpollinating “cheater” congener. Estimates assume equal sex ratios in figs where females of both *Eupristina* species were present

Study site	Pollinator prevalence (%)	Pollinator abundance in figs it occupied (*n* figs)	Female pollinator abundance in figs it occupied (*n* figs)	Cheater prevalence (%)	Cheater abundance (*n* figs)	Seed prevalence (%)	*N* seeds (*n* figs)
Mianyang	0	–	–	–	–	0	–
Chengdu	30.0	6.3 ± 1.2 (6)	4.0 ± 0.7 (6)	–	–	30.0	8.2 ± 1.6 (6)
Xichang	1.7	10.0 ± 3.9 (3)	4.0 ± 1.5 (3)	–	–	2.8	6.4 ± 1.0 (5)
Panzhihua	12.6	30.6 ± 4.0 (16)	24.6 ± 5.6 (16)	–	–	11.8	23.9 ± 2.8 (15)
Kunming	0	–	–	12.1	11.3 ± 4.5 (4)	6.1	1.5 ± 0.7 (2)
Xishuangbanna	68.9	32.4 ± 2.9 (111)	29.2 ± 2.9 (111)	82.6	71.0 ± 4.1 (133)	72.7	28.6 ± 3.2 (117)
Overall	24.5	30.6 ± 2.7 (136)	27.0 ± 2.5 (136)	24.7	69.3 ± 4.1 (137)	26.1	26.1 ± 2.8 (145)

The cheater *Eupristina* species was found only in the figs from Kunming and Xishuangbanna, where it occupied 12.1% and 82.6% of the figs, respectively (Table4). In a very small number of figs, it was recorded as an “accidental” pollinator, with 50.0% (2 of 4 figs in Kunming) and 19.6% (9 of 46 figs in Xishuangbanna) of the figs that contained offspring of this species (but not *E. verticillata*) also containing small numbers of seeds.

### Latitudinal and altitudinal effects on fig wasp community

**Figure 4:**
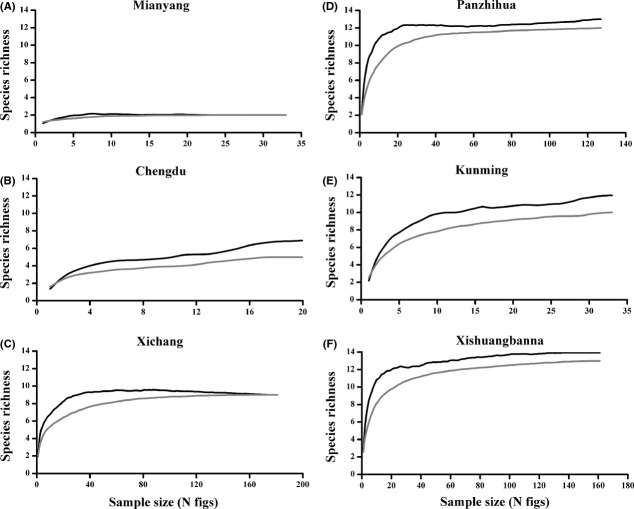
Estimated *Ficus microcarpa* fig wasp regional species richness in relation to sample sizes at six sites in southwest China (Mian Yang (A), Chengdu (B), Xichang (C), Panzhihua (D), Kunming (E) and Xishuangbanna (F)). Black lines indicate estimated species accumulations using a first-order jackknife algorithm, and gray lines are observed values.

We recorded 21 fig wasp morphospecies from the figs of *F. microcarpa* in southwest China (Table5), but no more than 13 species were recorded from any individual site (Tables2 and 5). Xishuangbanna had several species that were not recorded elsewhere, but there were also other species that were only recorded at other sites. Although several species of phytophages and their associated parasitoids were only recorded at intermediate latitudes, no clearly northern species were present (Table5). Both direct accumulation and first-order jackknife methods suggest that regional species richness almost reached asymptotes within our range of sample sizes at every site (Fig.4) and estimates of the size of the regional pools from which our samples were drawn suggest that we had recorded most but not all of the species predicted to be present at each site (Table2).

**Table 5:** Distributions of fig wasp species in *Ficus microcarpa* figs. The species abbreviations: *Ev, Eupristina verticillata* Waterston (Pollinator); *Es, Eupristina* sp. (cheater); *Aq, Acophila quinata* Zhang & Xiao; *Mb, Meselatus bicolor* Chen; *Oc, Odontofroggatia corneri* Wiebes; *Og, Odontofroggatia galili* Wiebes; *Oi, Odontofroggatia ishii* Wiebes; *Sbs, Sycobia* sp.; *Md, Micranisa degastris* Chen; *Wm, Walkerella microcarpae* Bouček; *Wn, Walkerella nigrabdomina* Ma & Yang; *Pt, Philotrypesis taiwanensis* Chen; *Bs, Bruchophagus sensoriae* Chen; *Sm, Sycophila maculafacies* Chen (“dark”); *Smp, Sycophila maculafacies* Chen (“pale”); *Sp, Sycophila petiolata* Chen; *Os, Ormyrus* sp.; *Pe, Philotrypesis emeryi* Grandi; *Po, Philotrypesis okinavensis* Ishii; *Srm, Sycoryctes moneres* Chen; *Scg, Sycoscapter gajimaru* Ishii

Trophic level	Phytophages (gallers and seed predator)											
Family (subfamily)	Agaonidae (Agaoninae)	Pteromalidae (Epichrysomallinae)						Pteromalidae (Otitesellinae)			Pteromalidae (Sycoryctinae)
Morphospecies	*Ev*	*Es*	*Aq*	*Mb*	*Oc*	*Og*	*Oi*	*Sbs*	*Md*	*Wm*	*Wn*	*Pt*
Mianyang				√	√							
Chengdu	√			√	√	√				√		
Xichang	√			√	√	√		√		√		
Panzhihua	√			√	√	√		√		√		√
Kunming		√	√	√	√				√	√		
Xishuangbanna	√	√	√		√		√		√	√	√	√

Trophic level	Parasitoids								
Family (subfamily)	Eurytomidae				Ormyridae	Pteromalidae (Sycoryctinae)			
Morphospecies	*Bs*	*Sm*	*Smp*	*Sp*	*Os*	*Pe*	*Po*	*Srm*	*Scg*
Mianyang									
Chengdu									
Xichang		√	√	√					
Panzhihua	√	√	√	√			√		
Kunming	√	√			√		√		
Xishuangbanna		√				√		√	√

√, Present.

Xishuangbanna supported the highest fig wasp species richness (Table2). There was a latitudinal shift in the character of the fig wasp communities, with a northwards decline in the abundance of the agaonids and their associated parasitoids and an increasing preponderance of fig wasps that make larger galls such as *Meselatus*, *Odontofroggatia*, and *Walkerella* species, together with their associated parasitoids (mainly *Sycophila maculafacies* and *Philotrypesis okinavensis sensu* Chen et al. (1999); Table S2). Mean species richness per fig also declined significantly with latitude (Tables2 and 3; Fig. S1), but did not exceed three species per fig, even at the southerly sites, despite many more species in total being recorded there.

Diversity, as measured by the Shannon–Wiener index, was highest at intermediate latitudes. In the two most northerly sites, Mianyang and Chengdu, this reflected the low species richness, while in the south, at Xishuangbanna, species richness was high, but many species were rare and offspring of the two *Eupristina* agaonids predominated, occupying over 97% of the figs and comprising over 91% of all fig wasps (Tables4 and S2). Despite this pattern, there was a significant decline in the Shannon–Wiener index with increasing latitude (Table3; Fig.3C).

Altitude was negatively correlated with ovule occupancy rate, fig wasp abundance, and species richness, and was positively related to Shannon–Wiener index values, suggesting altitude is also playing an important role in shaping the fig wasp fauna (Table3; Fig.3D–F). We failed to detect any significant effects of the interaction between latitude and altitude (Table S3).

### Comparisons between trophic levels

The overall fig wasp community from the six sites combined included 12 phytophages and nine parasitoids (Table5). Generally, offspring of phytophagous species were far more abundant than those of parasitoid species, comprising 90.5% of the total number of fig wasp individuals in the figs (Table S4). There were no parasitoids present in the figs from the two most northerly sites.

**Figure 5:**
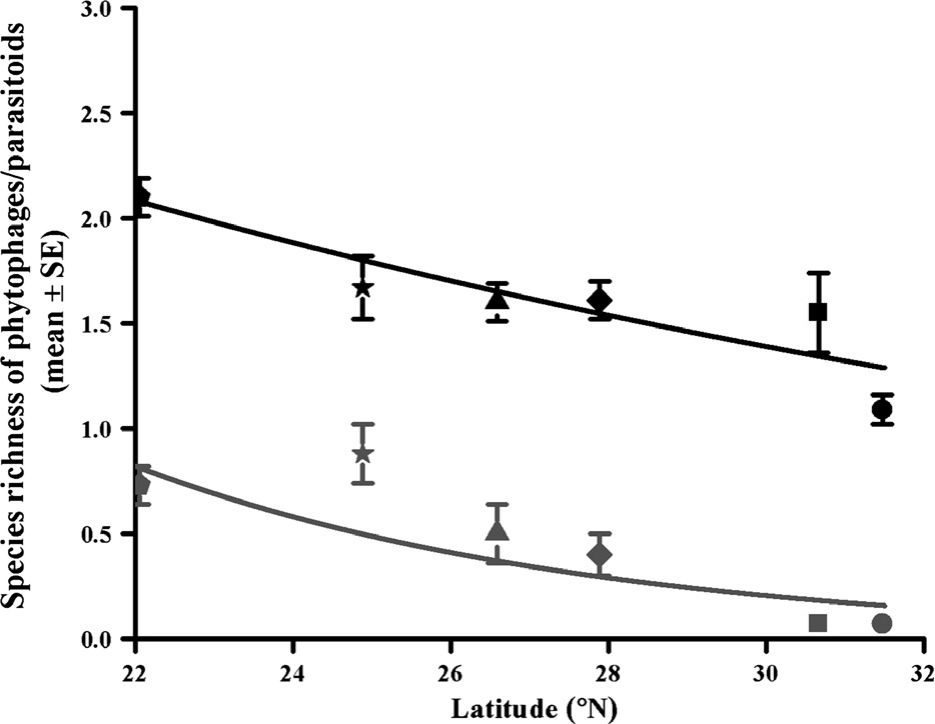
The numbers of phytophagous and parasitoid species in fig wasp communities associated with *Ficus microcarpa* in relation to latitude. GLMs assumed quasi-Poisson distributions of residuals. Black lines = phytophages and gray lines = parasitoids. Pentagons, stars, triangles, diamonds, squares, and circles indicate Xishuangbanna, Kunming, Panzhihua, Xichang, Chengdu, and Mianyang, respectively.

Figs from higher latitude sites contained significantly fewer species at both trophic levels, but parasitoid species declined significantly more rapidly with latitude than phytophagous species (Tables3 and S4; Fig.5). In contrast, altitude only had a significant influence on species richness of phytophagous species (Table3).

## Discussion

Apparent exceptions to global-scale declines in parasitoid species richness at higher latitudes may often be a result of sampling bias (Hawkins and Compton 1992; Sime and Brower 1998; Quicke 2012). Within more local communities, contrary species richness patterns have nonetheless been reported among gall wasp parasitoid assemblages in Canada (Bannerman et al. 2012) and fig wasps in South Africa (Hawkins and Compton 1992). The fig wasp community associated with *F. microcarpa* in southwest China, where planting has extended its range northwards by about 1000 km, changes in both species richness and composition with latitude. As would be expected, given that the range extension has probably only occurred within the last 100 years, there are no fig wasps restricted to higher latitudes, but some species were also rare or absent from the far-southern tropical forest environment of Xishuangbanna. The extent to which each fig wasp species has extended its range northwards beyond their host plant's original range is variable. Moving northward from the original range margin, the fall in species richness is also reflected in less intense utilization of the floral resources offered by the fig tree, and with parasitoid fig wasps declining more rapidly than exclusively phytophagous NPFW.

Individual figs from *F. microcarpa* trees growing at different latitudes offer identical resources to fig wasps, but the biotic and abiotic environments where the figs are offered are much more variable. Physiological tolerances among the fig wasps may have a significant role in determining their distributions, including their northerly range limits (Warren et al. 2010). Which stages of the insects’ life cycles are particularly climate sensitive are unclear, but low temperatures will influence larval development times, the ability of the adult offspring wasps to emerge from the figs, and their ability to migrate between trees to look for oviposition sites (Yang et al. 2013). Between-species variation in flexibility of development rates may be critical, because low winter temperatures at the more northern (and higher altitude) sites can extend the fig wasps’ development times from a few weeks to several months. The fruiting phenologies of fig trees are sensitive to local climate variables, especially temperature, with fewer figs produced and slower fig development rates during colder winter months, and initiation of new figs can stop entirely during the winter at cooler sites (Peng et al. 2010; Yang et al. 2013; Liu et al. 2014; Zhang et al. 2014).

Local climatic variables within the geographic range encompassed by our study sites were correlated more strongly with altitude than latitude, due to the large range in elevations. At Kunming, the higher altitude site located toward the southern edge of our study area, this is reflected in lower average minimum temperatures than the other sites, but also with relatively low mean maxima and less seasonal variation temperatures than at sites further north.

The dispersal abilities of different groups of fig wasps are unknown, but long-distance dispersal events (extending to 100 km or more) have been recorded in some species (Ahmed et al. 2009; Wang et al. 2009; Chen et al. 2012). Nevertheless, given that *F. microcarpa* is an introduced species at most of our study sites, and we know of no examples of the plant establishing itself outside of urban areas, there must be large gaps between the plant's introduced populations, some of which may be beyond the dispersal limits of most fig wasp species and result in isolation by distance effects contributing to the northerly decline in community complexity.

Some postulated reasons for global declines in parasitoid species richness with latitude, such as a lack of alternative hosts at more northerly sites, can be rejected because most fig wasps associated with *F. microcarpa* appear to be host plant specific (R. Wang & S.G. Compton, unpubl.). The more pronounced northerly decline in species richness and abundance among parasitoid NPFW appears to be linked to a shortage of hosts, rather than their complete absence. At higher latitudes, potential hosts were present at lower densities both within individual figs and in terms of the proportion of the figs that contained any fig wasps. Differences in the dispersal ability of phytophagous and parasitoid NPFW could also contribute if some species go extinct locally each winter at higher latitude sites and there are annual rescue effects from populations further south (Bannerman et al. 2012). The relative dispersal ability of different groups of fig wasps is unknown, but it is possible that dispersal among parasitoid species is hampered by the long ovipositors possessed by many species. These allow the parasitoids to lay their eggs through the walls of figs, but seem likely to reduce flight efficiency. Some parasitoid fig wasps with long ovipositors are nonetheless able to colonize even very isolated desert fig trees in Africa (Ahmed et al. 2009).

Planted *F. microcarpa* trees are capable of surviving beyond the natural range limit of the species, suggesting that there are germination and establishment issues that limited the tree's distribution in the past. From the tree's perspective, individuals planted further north also increasingly produced figs that were of no reproductive value, because they were seldom or never colonized by pollinators and were only colonized by gall-forming NPFW. Seed production was therefore limited or absent. The monoecious fig tree with a natural distribution that extends furthest north in China, *F. virens,* also struggles to support populations of its pollinator fig wasp through the winter at its northern range limit, but seed production is supported by the seasonal migration of pollinators from further south (Zhang et al. 2014). *F. virens* is pollinated by a species of *Platyscapa*, a genus where long-distance pollinator dispersal may be the norm (Burnham and Overton 1978). The *E. verticillata* pollinator of *F. microcarpa* may be less mobile and also more sensitive to temperature effects than *Platyscapa* sp.

*Eupristina verticillata* was absent from the southerly, but high altitude, Kunming study site, which experiences low mean monthly temperatures. It was replaced there by an abundant second (“cheater”) *Eupristina* species, which had a distribution limited to our two most southern study sites. Why this species should be restricted to the south, but apparently thrive at high altitude, is unclear. The two *Eupristina* species coexist at the lowland Xishuangbanna study site, but at Kunming *Eupristina* sp. replaced the pollinator in the figs, and the host plant largely failed to set seed there. Localized competitive exclusion involving congeneric fig wasps has been reported from another fig tree species (Liu et al. 2014) although the same mechanism of exclusion is unlikely to be present in *F. microcarpa*. Whatever the relative significance of competitive and environmental factors in the absence of the pollinator from Kunming, the results from this high altitude site emphasize the complexity of factors influencing the composition of fig wasp communities that colonize fig trees planted outside their natural range.

## Supporting Information

Figure S1. Frequency distributions of *Ficus microcarpa* fig wasp species richness per fig at six sites in SW China, ordered from north to south. **Table S1.** The first and second principal components of climate factors using Principal Component Analysis (PCA). **Table S2.** The most abundant fig wasp species at each study site. **Table S3.** Linear and generalized linear models examining aspects of fig wasp community composition in relation to the interaction between latitude and altitude (Latitude × Altitude; only figs that contained fig wasps are included). **Table S4.** The species richness and abundance of phytophagous and parasitoid fig wasps in *F. microcarpa* figs (only figs that contained fig wasps are considered).
