# Supplementary material for: Insect responses to host plant provision beyond natural boundaries: latitudinal and altitudinal variation in a Chinese fig wasp community
Source: Ecol Evol. 2015 Aug 13;5(17):3642–56. doi: 10.1002/ece3.1622 (PMC4567868; doi:10.1002/ece3.1622)
Supplement: Figure S1. — Frequency distributions of Ficus microcarpa fig wasp species richness per fig at six sites in SW China, ordered from north to south. Table S1. The first and second principal components of climate factors using Principal Component Analysis (PCA). Table S2. The most abundant fig wasp species at each study site. Table S3. Linear and generalized linear models examining aspects of fig wasp community composition in relation to the interaction between latitude and altitude (Latitude × Altitude; only figs that contained fig wasps are included). Table S4. The species richness and abundance of phytophagous and parasitoid fig wasps in F. microcarpa figs (only figs that contained fig wasps are considered). [file ece30005-3642-sd1.docx]

**Table S1.** The first and second principal components of climate factors using Principal Component Analysis (PCA).

|  | PC1 | PC2 |
| --- | --- | --- |
| Standard deviation | 2.212 | 1.273 |
| Proportion of Variance | 0.611 | 0.203 |
| Cumulative Proportion | 0.611 | 0.814 |
| Average temperature | -0.415 | 0.292 |
| Winter average temperature | -0.303 | 0.580 |
| Winter average low temperature | -0.417 | 0.425 |
| Summer average temperature | -0.399 | -0.356 |
| Summer average high temperature | -0.386 | -0.392 |
| Annual precipitation | -0.407 | -0.340 |
| Winter precipitation | -0.094 | -0.082 |
| Summer precipitation | -0.332 | -0.136 |

**Table S2.** The most abundant fig wasp species at each study site. Only figs that contained fig wasps are considered. Sites are ordered from north-south. Sample sizes are given in Table 2. Males of pollinators were estimated using the sex ratio for total agaonids in figs containing females of both *Eupristina* species.

| Trophic level | Study site | Predominant taxon | Prevalence  (% figs occupied) | Abundance in occupied figs  Mean ± SE (n figs) | Relative abundance (% of all fig wasps at the site) |
| --- | --- | --- | --- | --- | --- |
| Phytophagous |  |  |  |  |  |
|  | Mianyang | *M. bicolor* | 93.9 | 21.9 ± 2.4 (31) | 97.4 |
|  | Chengdu | *O. galili* | 65.0 | 10.3 ± 2.3 (13) | 63.5 |
|  | Xichang | *O. corneri* | 66.3 | 9.6 ± 0.8 (120) | 37.9 |
|  | Panzhihua | *W. microcarpae* | 65.4 | 7.9 ± 0.9 (83) | 27.2 |
|  | Kunming | *O. corneri* | 63.6 | 13.0 ± 3.6 (21) | 31.2 |
|  | Xishuangbanna | *Eupristina* cheater | 82.6 | 71.0 ± 4.1(133) | 66.3 |
| Parasitoids |  |  |  |  |  |
|  | Mianyang | None present |  |  |  |
|  | Chengdu | None present |  |  |  |
|  | Xichang | *S. maculafacies* | 30.4 | 9.4 ± 0.7 (55) | 17.0 |
|  | Panzhihua | *P. okinavensis* | 26.8 | 7.9 ± 0.7 (34) | 11.0 |
|  | Kunming | *P. okinavensis* | 45.5 | 11.4 ± 3.0 (15) | 19.5 |
|  | Xishuangbanna | *S. gajimaru* | 31.7 | 6.7 ± 4.5 (51) | 2.4 |

**Table S3.** Linear and generalized linear models examining aspects of fig wasp community composition in relation to the interaction between latitude and altitude (Latitude × Altitude; only figs that contained fig wasps are included). LR = likelihood ratio. Response variables are:

(1) occupancy rates (the proportion of female flowers that supported fig wasp adult offspring, calculated with or without inclusion of figs that contained *Meselatus bicolor* (MB)),

(2) fig wasp abundance (the numbers of wasps present in figs occupied by fig wasps),

(3) Shannon-Wiener diversity index (S-W index),

(4) fig wasp species richness (SR),

(5) phytophagous fig wasp species richness (GSR),

(6) parasitoid fig wasp species richness (PSR).

| Fixed effect | Response variables | Model | Residuals | df | LR |
| --- | --- | --- | --- | --- | --- |
| Latitude × Altitude | | | | | |
|  | Occupancy rate | GLM | Quasibinomial | 1 | 0.56 ^NS^ |
|  | Occupancy rate (no MB) | GLM | Quasibinomial | 1 | 62.69 ^NS^ |
|  | Fig wasp abundance | GLM | Quasibinomial | 1 | 3.17 ^NS^ |
|  | SR | GLM | Quasibinomial | 1 | 0.26 ^NS^ |
|  | S-W index | LM | Normal | 1 | 0.76 ^NS^ |
|  | GSR | GLM | QuasiPoisson | 1 | 0.33 ^NS^ |
|  | PSR | GLM | QuasiPoisson | 1 | 2.84 ^NS^ |

^NS^: not significant.

**Table S4.** The species richness and abundance of phytophagous and parasitoid fig wasps in *F. microcarpa* figs (only figs that contained fig wasps are considered). S (obs) = number of species recorded. Means ± S.E (per fig) are presented. Abundance of phytophages and parasitoids was based on all figs occupied by fig wasps, including those where parasitoids were absent.

| Study site | S (obs) phytophages | S (obs) parasitoids | Phytophage species richness | Parasitoid  Species richness | Parasitoid species: phytophagous species ratios | Phytophages abundance | Parasitoids abundance |
| --- | --- | --- | --- | --- | --- | --- | --- |
| Mianyang | 2 | 0 | 1.09 ± 0.05 | 0 | 0 | 21.1 ± 2.2 | 0 |
| Chengdu | 5 | 0 | 1.55 ± 0.17 | 0 | 0 | 10.6 ± 1.6 | 0 |
| Xichang | 6 | 3 | 1.61 ± 0.05 | 0.41 ± 0.04 | 0.32 ± 0.03 | 12.9 ± 0.8 | 3.9 ± 0.5 |
| Panzhihua | 7 | 5 | 1.60 ± 0.06 | 0.52 ± 0.07 | 0.37 ± 0.05 | 15.7 ± 1.3 | 3.4 ± 0.5 |
| Kunming | 6 | 4 | 1.67 ± 0.13 | 0.88 ± 0.12 | 0.60 ± 0.09 | 17.3 ± 3.5 | 7.8 ± 1.8 |
| Xishuangbanna | 9 | 4 | 2.10 ± 0.07 | 0.65 ± 0.06 | 0.33 ± 0.04 | 84.5 ± 3.2 | 3.6 ± 0.5 |
| Overall | 12 | 9 | 1.72 ± 0.03 | 0.49 ± 0.03 | 0.32 ± 0.02 | 35.0 ± 1.7 | 3.5 ± 0.3 |





**Figure S1. Frequency distributions of *Ficus microcarpa* fig wasp species richness per fig at six sites in SW China, ordered from north to south.**
